# A method of extending the depth of focus of the high-resolution X-ray imaging system employing optical lens and scintillator: a phantom study

**DOI:** 10.1186/1475-925X-14-S1-S15

**Published:** 2015-01-09

**Authors:** Guang Li, Shouhua Luo, Yuling Yan, Ning Gu

**Affiliations:** 1 School of Biological Science and Medical Engineering, Southeast University, Nanjing 210009 China; 2 Department of Bioengineering, School of Engineering, Santa Clara University, CA 95053, USA

## Abstract

**Background:**

The high-resolution X-ray imaging system employing synchrotron radiation source, thin scintillator, optical lens and advanced CCD camera can achieve a resolution in the range of tens of nanometers to sub-micrometer. Based on this advantage, it can effectively image tissues, cells and many other small samples, especially the calcification in the vascular or in the glomerulus. In general, the thickness of the scintillator should be several micrometers or even within nanometers because it has a big relationship with the resolution. However, it is difficult to make the scintillator so thin, and additionally thin scintillator may greatly reduce the efficiency of collecting photons.

**Methods:**

In this paper, we propose an approach to extend the depth of focus (DOF) to solve these problems. We develop equation sets by deducing the relationship between the high-resolution image generated by the scintillator and the degraded blur image due to defect of focus first, and then we adopt projection onto convex sets (POCS) and total variation algorithm to get the solution of the equation sets and to recover the blur image.

**Results:**

By using a 20 *μm *thick unmatching scintillator to replace the 1 *μm *thick matching one, we simulated a high-resolution X-ray imaging system and got a degraded blur image. Based on the algorithm proposed, we recovered the blur image and the result in the experiment showed that the proposed algorithm has good performance on the recovery of image blur caused by unmatching thickness of scintillator.

**Conclusions:**

The method proposed is testified to be able to efficiently recover the degraded image due to defect of focus. But, the quality of the recovery image especially of the low contrast image depends on the noise level of the degraded blur image, so there is room for improving and the corresponding denoising algorithm is worthy for further study and discussion.

## Introduction

Currently, high-resolution X-ray imaging systems such as Nano-CT based on the third-generation synchrotron sources and X-ray detector with transparent luminescent screen have been widely used to achieve a high spatial resolution in sub-micrometer or nanometer range [1,2]. So it can be effectively used to image tissues, cells and many other small samples in-vitro or in-vivo, especially the calcification in the vascular and in the glomerulus. The detector consists of scintillator (YAG:Ce or LuAG:Ce), microscope optics and low-noise CCD. The scintillator receives the x-ray and converts it into visible light, which is then magnified by the microscope optics and collected by the CCD camera [2]. To achieve good image quality, the depth of focus of the microscope optics must match the thickness of the scintillator. The final spatial resolution depends both on the thickness of the scintillator and the depth of focus. According to Rayleigh criterion, the microscope lens in detection systems that employ transparent luminescent screens usually has a large numeric aperture (NA) to extend the limits of resolution of certain wavelengths of visible light. However, with an increasing NA, the depth of focus will become thinner and thinner. Therefore if we want to focus the lens into the thin layer of the luminescent screen and to obtain an image of good quality, we must grind the luminescent screen very thin even down to several micrometers or less. The thinning of the scintillator lowers the efficiency of converting X-ray into visible light and makes the manufacture challenging. For example, if we desire that the detection system employing transparent luminescent screens should achieve the limit of resolution of visible light (~0.3 *μm*), the thickness of corresponding scintillator should be ~0.5 *μm *according to Rayleigh criterion and formula of depth of focus. In the traditional microscope system, the resolution is proportional to 1/NA, and the depth of focus is proportional to 1/NA^2, so they are incompatible and usually we must compromise the two parameters to suit specific needs. Fortunately, in the detection system employing luminescent screens, there are some unique properties that we can employ to extend the depth of focus. One of them is everywhere in the scintillator is uniform, so the light intensity distribution across arbitrary layer in the scintillator is identical except the minor difference that may arise from the divergence of X-ray and the change in amplitude due to the x-ray attenuation. The divergence is negligible because the scintillator is relatively thin and the cone angle is very small for most high-resolution X-ray imaging systems [2]. For example, the scintillator installed before the objective with 5× magnification is about 20 *μm *thick, and the one matching with 20× objective lens is about 5 *μm*. And usually in those cases, the effect of divergence can not be seen. In order to avoid the effect of divergence, we just focus our method on extending the depth of focus of objective lens with higher magnification than 5× to about 20 *μm*. Because the 20 *μm*-thick scintillator is easy to process and if we can use it to take place of the much thinner scintillator needed in the higher resolution X-ray imaging systems, we can greatly improve the resolution without considering the influence of the defect of focus. Our method is similar to light field imaging [3,4] but without any other additional facilities. In the experiment, we first simulated the continuous fluorescencing process of the scintillator, and approximately got a composite point spread function model by using one property that point spread function of a certain luminous plane of a microscopic system follows Gaussian distribution. Subsequently, we simulated a blur projection image resulted from a little thicker scintillator which is not matching with the magnification scale of the optical lens. After that, we adopted POCS and total variation algorithm to recover the blur image. The results in the experiment show that we can effectively extend the depth of focus of the high-resolution X-ray imaging systems by using this algorithm.

## Methods

### Simulated imaging

**Figure 1:**
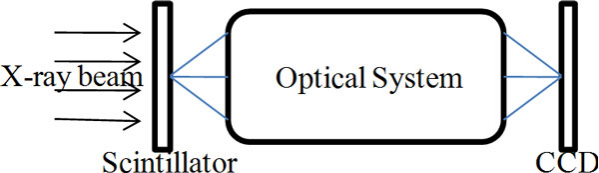
**X-ray imaging system model employing transparent luminescent screen and optical system**.

**Figure 2:**
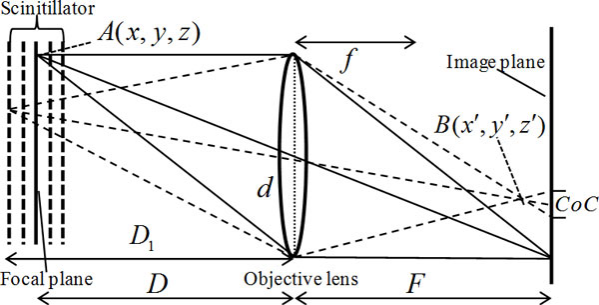
**Imaging model of high-resolution X-ray imaging system in which the thickness of the employed scintillator is not matching with the objective lens**. D: Object distance, F: Image plane distance, d: Diameter of lens aperture, f: Focal length, CoC: Circle of Confusion, A(x,y,z):Object point, B(x',y',z'):virtual imaging point.

In the high-resolution X-ray imaging system, the detector consists of thin scintillator material, optical lens with magnification and low noise CCD camera (Figure 1). The thickness of the scintillator should be matched with the depth of focus of the objective lens, e.g., one objective with 20× magnification and resolution of 1 *μm*, its DOF is about 5 *μm *while the thickness of the scintillator would be 5 *μm *or less. When the scintillator is thicker than that, a point on some layer of the scintillator that is away from the focal plane is focused before or behind the sensor plane. This point becomes a circle called circle of confusion (CoC) in the sensor, just as shown in Figure 2. When this circle is smaller than a pixel, it cannot be seen, and that is why depth of focus exists. But when the thickness of the scintillator exceeds the depth of focus, all the circles of confusion from different planes will overlap and make the resultant image blur. The size of CoC can be derived from the basic geometric relationship among image plane, object plane and focal length, and this relationship can be expressed as Eq. (1).

(1) $$\frac{1}{D}+\frac{1}{F}=\frac{1}{f}.$$

Here, D is object distance, F is the conjugate distance of D and f is the focal length. The direction z is parallel to the optical axis. According to the imaging equation, object plane at the distance of D_1 _is focused before the immediate plane. It becomes CoC in the immediate plane. The diameter of the circle can be calculated by Eq. (2).

(2) $$d_{CoC}=\left|\frac{D}{D_{1}}\cdot \frac{\left(D_{1}-f\right)}{\left(D-f\right)}-1\right|\cdot d.$$

Here, *d *is the diameter of the aperture lens. When *d_CoC _*is bigger than the pixel size of the image sensor, the resolution of the image captured will decrease.

In the usual optical lens systems, image blur can be considered as the degradation model with a convolution filter *h*(*x*, *y*) and this model can be denoted by

(3) g(x,y)=h(x,y)*f(x,y)+n(x,y),

where, *h*(*x*, *y*) is the impulse response or point spread function (PSF) which is usually relative with CoC of the imaging system, *n*(*x*, *y*) is the additive noise and *g*(*x*, *y*) is the blur image. PSF of the defocused lens can be either modeled by the geometrical or physical optics [5]. The former ignores the effect of diffraction and it can be considered as uniform distribution when the aperture is circular. For the physical optics case, diffraction and aberration is taken into consideration and the signal intensity in the CoC is assumed to follow Gaussian distribution [6-8] as follow.

(4) $$h\left(x,y\right)=\frac{1}{2\pi \sigma^{2}}e^{-\frac{x^{2}+y^{2}}{2\sigma^{2}}}.$$

Here, the spread parameter *σ *is proportional to the radius of the CoC [6-8]. Because the radiuses of the CoC formed by different scintillator planes are not identical, the response functions of different planes are not same, and because the divergence can be neglected, the final blur image generated by the photons transmitting from all the planes of the scintillator can be described as a form of integration as Eq. (5).

(5) $$g\left(x,y\right)=\overset{Z_{1}}{\underset{-Z_{0}}{\int }}f\left(x,y,z\right)*h\left(x,y,z\right)dz=\overset{Z_{1}}{\underset{-Z_{0}}{\int }}e^{-\xi z}f\left(x,y\right)*h\left(x,y,z\right)dz,$$

where, *z*_0 _refers to the distance from the focal plane in the scintillator to the scintillator's left surface, *z*_1 _represents the distance from the focal plane to the right surface, and *ξ *represents X-ray absorption coefficient. When assuming the image formed by the photons which come from the focal plane and transmit through the optical lens to be $g_{0}\left(x,y\right)\text{ = }f\left(x,y\right)\text{*}h\left(x,y,0\right)\text{ = }f\left(x,y\right)*\delta \left(x,y\right)\text{ = }f\left(x,y\right)$, all other images formed by the photons coming from other scintillator planes can be expressed as follow.

(6) $$g_{\text{z}}\left(x,y\right)\text{ = }f\left(kx,ky\right)\text{*}h\left(kx,ky,z\right).$$

Here, k is the scale factor and can be written as k=F(D+z)FD=DD+z. Now the imaging formula of the final degraded blur image needed to be modifies as

(7) $$g\left(x,y\right)=\overset{Z_{1}}{\underset{-Z_{0}}{\int }}f\left(kx,ky,z\right)*h\left(kx,ky,z\right)dz=\overset{Z_{1}}{\underset{-Z_{0}}{\int }}e^{-\xi z}f\left(kx,ky\right)*h\left(kx,ky,z\right)dz.$$

Its Fourier transformation can be described as

(8) $$\text{G}\left(u,v\right)=\overset{Z_{1}}{\underset{-Z_{0}}{\int }}e^{-\xi z}\frac{1}{k^{4}}F\left(\frac{z+D}{D}u,\frac{z+D}{D}v\right)e^{-M^{2}\left(u^{2}+v^{2}\right)z^{2}}dz,$$

where, $M^{2}={\left(ca\frac{1}{D}\right)}^{2},a=\frac{fd}{D-f}$ and c is a constant which is relative with the system. Now we can discretize G(*u*, *v*) and get G(m, n) as stated below.

(9) $$\begin{array}{c} G(m,n)=\overset{Z_{1}}{\underset{-Z_{0}}{\int^{\text{​}}}}e^{-\xi z}\frac{1}{k^{4}}F(\frac{z+D}{D}m\Omega ,\frac{z+D}{D}n\Omega )e^{-M^{2}\Omega^{2}(m^{2}+n^{2})z^{2}}dz\\ \;=\overset{Z_{1}}{\underset{-Z_{0}}{\int^{\text{​}}}}F(m\Omega \;+\;\frac{zm}{D}\Omega ,n\Omega +\frac{zn}{D}\Omega )e^{-M^{2}\Omega^{2}(m^{2}+n^{2})z^{2}-\xi z}\frac{1}{k^{4}}dz \end{array}$$

When $\lambda_{m}\leq \frac{zm}{D}<\lambda_{m}+1,\lambda_{n}\leq \frac{zn}{D}<\lambda_{n}+1$, and *λ_m_*, *λ_n _*are integers, we can get the approximate expression of $F\left(m\Omega \text{ + }\frac{zm}{D}\Omega ,n\Omega +\frac{zn}{D}\Omega \right)$ by using bilinear interpolation or nearest neighbor interpolation if *z *is quite small comparing with D. In our discussion, we adopt bilinear interpolation for more accuracy and assume that *z*_1 _is 10 *μm*, -*z*_0 _is -10 *μm*, D = 50 mm, sampling account of the blur image is 512*512, so $-0.1024\leq \frac{zm}{D}\leq 0.1024$, $-0.1024\leq \frac{zn}{D}\leq 0.1024$, and G(*m*, *n*) can approximately be transformed as

(10) $$\begin{array}{c} G\left(m,n\right)=F\left(m,n\right)\Psi_{m,n}+F\left(m,n+1\right)\Psi_{m,n+1}+F\left(m+1,n\right)\Psi_{m+1,n}+\\ \;F\left(m+1,n+1\right)\Psi_{m+1,n+1}\text{ + }F\left(m\text{ - }1,n\text{ - }1\right)\Psi_{m\text{ - }1,n\text{ - }1}+F\left(m\text{ - }1,n\right)\Psi_{m\text{ - }1,n}+\\ \;F\left(m,n\text{ - }1\right)\Psi_{m,n\text{ - }1} \end{array}$$

where,

$\Psi_{m,n}=\overset{Z_{1}}{\underset{0}{\int }}\left(1-\frac{z\left(m+n\right)}{D}+\frac{z^{2}mn}{D^{2}}\right)g\left(z\right)dz+\overset{0}{\underset{\text{ - }Z_{0}}{\int }}\left(1\text{ + }\frac{z\left(m+n\right)}{D}+\frac{z^{2}mn}{D^{2}}\right)g\left(z\right)dz$, $\Psi_{m,n+1}=\overset{Z_{1}}{\underset{0}{\int }}\left(\frac{zn}{D}-\frac{z^{2}mn}{D^{2}}\right)g\left(z\right)dz$,$\Psi_{m+1,n}\text{ = }\overset{Z_{1}}{\underset{0}{\int }}\left(\frac{zm}{D}-\frac{z^{2}mn}{D^{2}}\right)g\left(z\right)dz$,$\Psi_{m+1,n+1}\text{ = }\overset{Z_{1}}{\underset{0}{\int }}\frac{z^{2}mn}{D^{2}}g\left(z\right)dz$,$\Psi_{m\text{ - }1,n\text{ - }1}\text{ = }\overset{0}{\underset{\text{ - }Z_{0}}{\int }}\frac{z^{2}mn}{D^{2}}g\left(z\right)dz$,$\Psi_{m\text{ - }1,n}\text{ = }\overset{0}{\underset{\text{ - }Z_{0}}{\int }}\left(-\frac{zm}{D}-\frac{z^{2}mn}{D^{2}}\right)g\left(z\right)dz$ and $\Psi_{m,n\text{ - }1}\text{ = }\overset{0}{\underset{\text{ - }Z_{0}}{\int }}\left(-\frac{zn}{D}-\frac{z^{2}mn}{D^{2}}\right)g\left(z\right)dz$, and $e^{-M^{2}\Omega^{2}\left(m^{2}+n^{2}\right)z^{2}-\xi z}\frac{1}{k^{4}}\text{ = g}\left(z\right)$.

If we set *ψ *to be the inverse transform of function Ψ, the expression of the blur image in spatial domain can be expressed as

(11) $$\begin{array}{c} \text{g}\left(k,l\right)\text{ = }f\left(k,l\right)\text{*}\psi_{m,n}+\left(f\left(k,l\right)W_{N}^{l}\right)*\psi_{m,n+1}+\left(f\left(k,l\right)W_{M}^{k}\right)*\psi_{m+1,n}+\\ \;(f\left(k,l\right)W_{M}^{k}W_{N}^{l})*\psi_{m+1,n+1}+\left(f\left(k,l\right)W_{M}^{\text{ - k}}W_{N}^{-l}\right)*\psi_{m-1,n-1}+\\ \left(f\left(k,l\right)W_{M}^{-k}\right)*\psi_{m-1,n}+\left(f\left(k,l\right)W_{N}^{-l}\right)*\psi_{m,n-1} \end{array}$$.

where, $W_{N}^{l}\text{ = }e^{-j\frac{2\pi }{N}l}$. For now, we set up a relationship between the image *f*(*k*, *l*) generated by the luminous plane located on the focal plane and the blur image *g*(*k*, *l*).

(12) $$\overset{M}{\underset{i=0}{\sum }}\overset{N}{\underset{j=0}{\sum }}w_{i,j,k,l}f\left(i,j\right)=g\left(k,l\right).$$

Here,

$$\begin{array}{c} w_{i,j,k,l}\text{ = }\psi_{m,n}\left(k-i,l-j\right)+W_{N}^{j}\psi_{m,n+1}\left(k-i,l-j\right)+W_{M}^{i}\psi_{m+1,n}\left(k-i,l-j\right)+W_{M}^{i}W_{N}^{j}\psi_{m+1,n+1}\left(k-i,l-j\right)\\ +W_{M}^{-i}W_{N}^{-j}\psi_{m-1,n-1}\left(k-i,l-j\right)+W_{M}^{-i}\psi_{m-1,n}\left(k-i,l-j\right)+W_{N}^{-j}\psi_{m,n-1}\left(k-i,l-j\right) \end{array}$$

and we will use this relationship to simulate the degraded blur image.

### Recovery algorithm

When the pixel number of the detector is small, it is easy to invert the system of equations by conventional matrix theory. However, in practice there are usually as many as tens of thousands of pixels in one detector, so it precludes any possibility to invert the matrix directly. For solving the problems with large numbers of functions, there is an attractive iterative method for solving them. That is called projection onto convex sets algorithm, which was developed by Kaczmarz [9] and can be briefly described as follows.

(13) $${\vec{f}}^{\left(\kappa \right)}={\vec{f}}^{\left(\kappa -1\right)}-\frac{\left({\vec{f}}^{\left(\kappa -1\right)}\cdot {\vec{w}}_{i}-p\left(i\right)\right)}{{\vec{w}}_{i}\cdot {\vec{w}}_{i}}{\vec{w}}_{i}.$$

The vector ${\vec{f}}^{\left(\kappa \right)}$ in Eq. (13) is composed of all the variables $f^{\left(k\right)}\left(1\right),f^{\left(k\right)}\left(2\right),\ldots ,f^{\left(k\right)}\left(M\right)$ that are generated by the kth iteration, and ${\vec{w}}_{i}=\left(w_{i1},w_{i2},\cdot \cdot \cdot ,w_{iM}\right)$ is the weighting vector. In our model, we have a similar form as described below.

(14) $$\{\begin{array}{l} {\vec{f}}^{\left(\kappa \right)}={\vec{f}}^{\left(\kappa -1\right)}-\frac{\left({\vec{f}}^{\left(\kappa -1\right)}\cdot {\vec{W}}_{k,l}-p(k,l)\right)}{{\vec{W}}_{k,l}\cdot {\vec{W}}_{k,l}}{\vec{W}}_{k,l}\\ {\vec{f}}^{\left(\kappa \right)}=(f(0,0),f(0,1),\cdots ,f(i,j),\cdots )\\ {\vec{W}}_{k,l}=(w_{0,0,k,l},w_{0,1,k,l},\cdots ,w_{i,j,k,l},\cdots ) \end{array}$$

By using the POCS algorithm we can basically solve the equation sets. However, since various types of noises and errors may exist during the imaging procedure, an accurate unique solution to the system of equations may not exist. Rather we can only obtain a near solution. Hence, the restored image is degenerated to some extent. In order to improve the quality of the restored image, we adopt a compress-sensing algorithm [10] based on the minimization of the image total variation (TV) during the solution procedure. The TV algorithm is first developed for signal noise reduction, and recently brought in CT reconstruction for insufficient data problems [11-13]. In this paper we construct a TV expression similar to the one in CT reconstruction. Combining above iterative algorithm with the TV regularization, the new solution procedure can be viewed as an optimization problem as stated below.

(15) $$\left\{\begin{array}{c} \text{min}||\vec{f}|{|}_{TV}\\ \overset{M}{\underset{i=0}{\sum }}\overset{N}{\underset{j=0}{\sum }}w_{i,j,k,l}f\left(i,j\right)=g\left(k,l\right)\\ f\left(i,j\right)\geq 0 \end{array}\right).$$

In Eq. (15), the vector $\vec{f}$ represents an image and is composed of *f*(*i*, *j*) and the expression $||\vec{f}|{|}_{TV}$ means the *l*_1 _norm of the gradient of the image represented by $\vec{f}$. The latter two parts of Eq. (15) are treated as data consistency constraint and positive constraint, and the former part is a regularization term. The TV expression can be described as

(16) $$||\vec{f}|{|}_{TV}=\iint \sqrt{||\nabla f\left(x,y\right)|{|}_{2}^{2}+\varepsilon^{2}}dxdy=\iint \sqrt{{\left(\frac{\partial f\left(x,y\right)}{\partial x}\right)}^{2}+{\left(\frac{\partial f\left(x,y\right)}{\partial y}\right)}^{2}+\varepsilon^{2}}dxdy$$

where *ε *is a small positive number. The minimization of TV of the image estimate can be achieved using many optimization algorithms such as gradient descent or conjugate gradient method. In this paper we choose the gradient descent method to search the minimization point that agrees with data consistency constraint. And the derivative of $||\vec{f}|{|}_{TV}$ can be expressed as an approximate discrete form as

(17) $$\begin{array}{c} \frac{\partial ||\vec{f}|{|}_{TV}}{\partial f\left(i,j\right)}=\frac{f\left(i,j\right)-f\left(i-1,j\right)}{\sqrt{{\left(f\left(i,j\right)-f\left(i-1,j\right)\right)}^{2}+{\left(f\left(i-1,j+1\right)-f\left(i-1,j\right)\right)}^{2}+\varepsilon^{2}}}+\\ \frac{f\left(i,j\right)-f\left(i,j-1\right)}{\sqrt{{\left(f\left(i+1,j-1\right)-f\left(i,j-1\right)\right)}^{2}+{\left(f\left(i,j\right)-f\left(i,j-1\right)\right)}^{2}+\varepsilon^{2}}}-\\ \frac{f\left(i+1,j\right)+f\left(i,j+1\right)-2f\left(i,j\right)}{\sqrt{{\left(f\left(i+1,j\right)-f\left(i,j\right)\right)}^{2}+{\left(f\left(i,j+1\right)-f\left(i,j\right)\right)}^{2}+\varepsilon^{2}}} \end{array}.$$

Here, we have specified all the parameters needed in the solution procedure. The solution procedure contains two phases: one is POCS and the other is TV gradient descent. The two phases are alternately executed iteratively. The POCS phase enforces the data consistency and the TV gradient descent phase reduces the TV of the intermediate image from the POCS phase.

## Experiment and result

**Figure 3:**
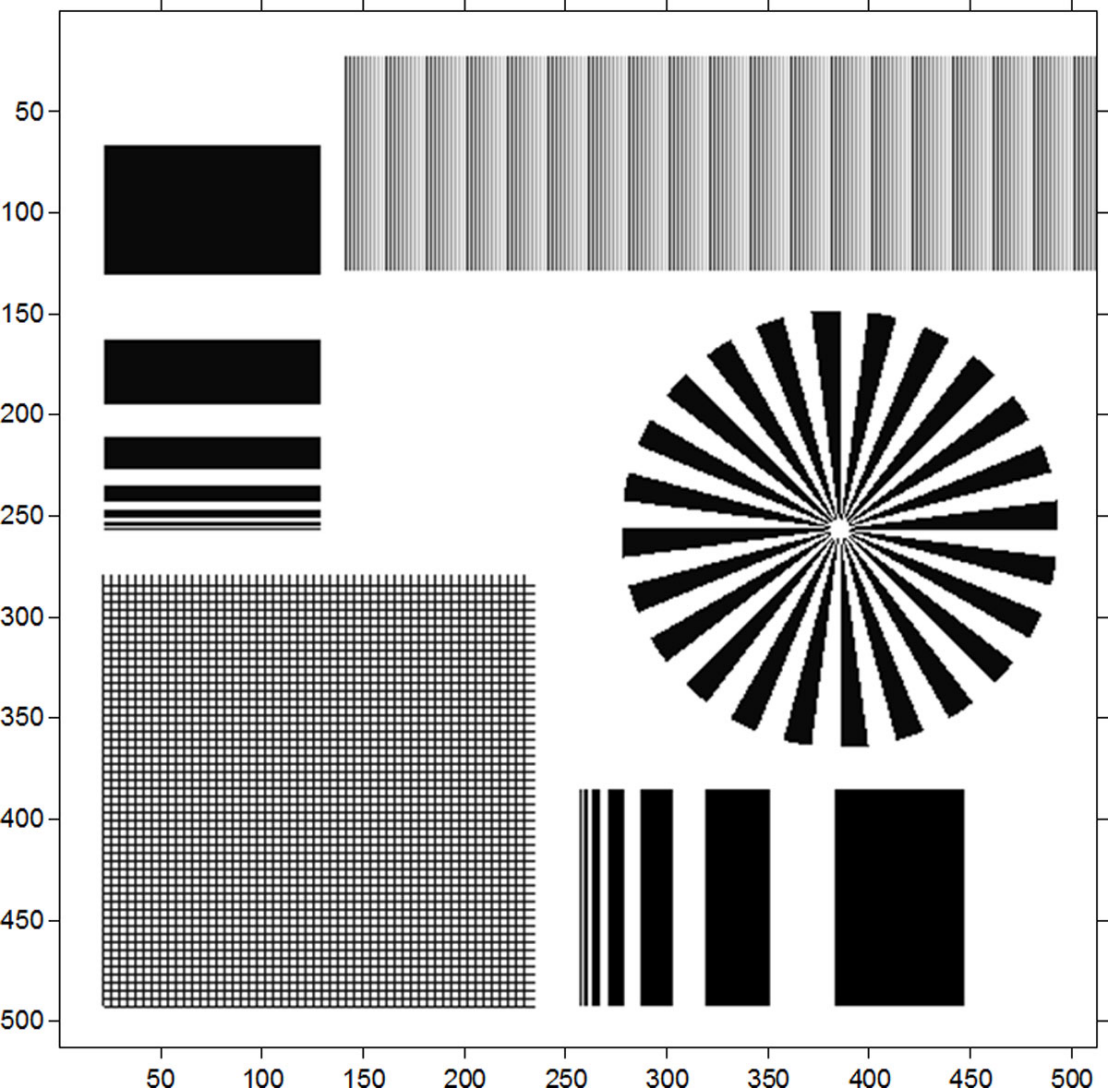
**The simulated resolution pattern which is used to test the effectiveness of the proposed algorithm**.

The simulated pattern (Figure 3) is 512*512 pixels in dimensions, and the pixel size is 1 *μm*. This pattern is composed of five parts and the finest feature size on it is 1 *μm*. The left top and the right bottom part contain a series of line pairs with different pitches from 1 *μm *to 128 *μm*. The left bottom part is a grid with pitch of 5 *μm*, and this grid together with the right middle Siemens star can be used to test the algorithm's performance on the complicated objects. The right top part contains line pairs with gradually varied grayscale ranging from 10 to 190. This part can be used to test the performance on the recovery of low contrast images. The specific parameters of the involved optical system are given in table 1. The focal length f is 40 mm. The object distance D is 50 mm and the image distance is 200 mm. The pixel size of detector is 1 *μm*, so the limited resolution of the optical system is 2 *μm *and the proper thickness of the scintillator should be about 1 *μm*. But the scintillator chosed in the experiment is 20 *μm *thick, and hence the resolution will greatly degrade. Given the parameters of the optical lens and the relationship between the degraded blur image and the original high-resolution image, we can approximately simulate a blur image caused by inappropriate thickness of scintillator and defect of focus. In the experiment, we assumed that the scintillator material was CsI and X-ray energy was 60 kV.

**Table 1:** Parameters of the simulated Optical System

Parameter	Value
f(Focal length)	40 mm
D(object distance)	50 mm
F(Sensor plane/image distance)	200 mm
d(Diameter of aperture)	25 mm
SPS(Sensor Pixel size)	0.001 mm

**Figure 4:**
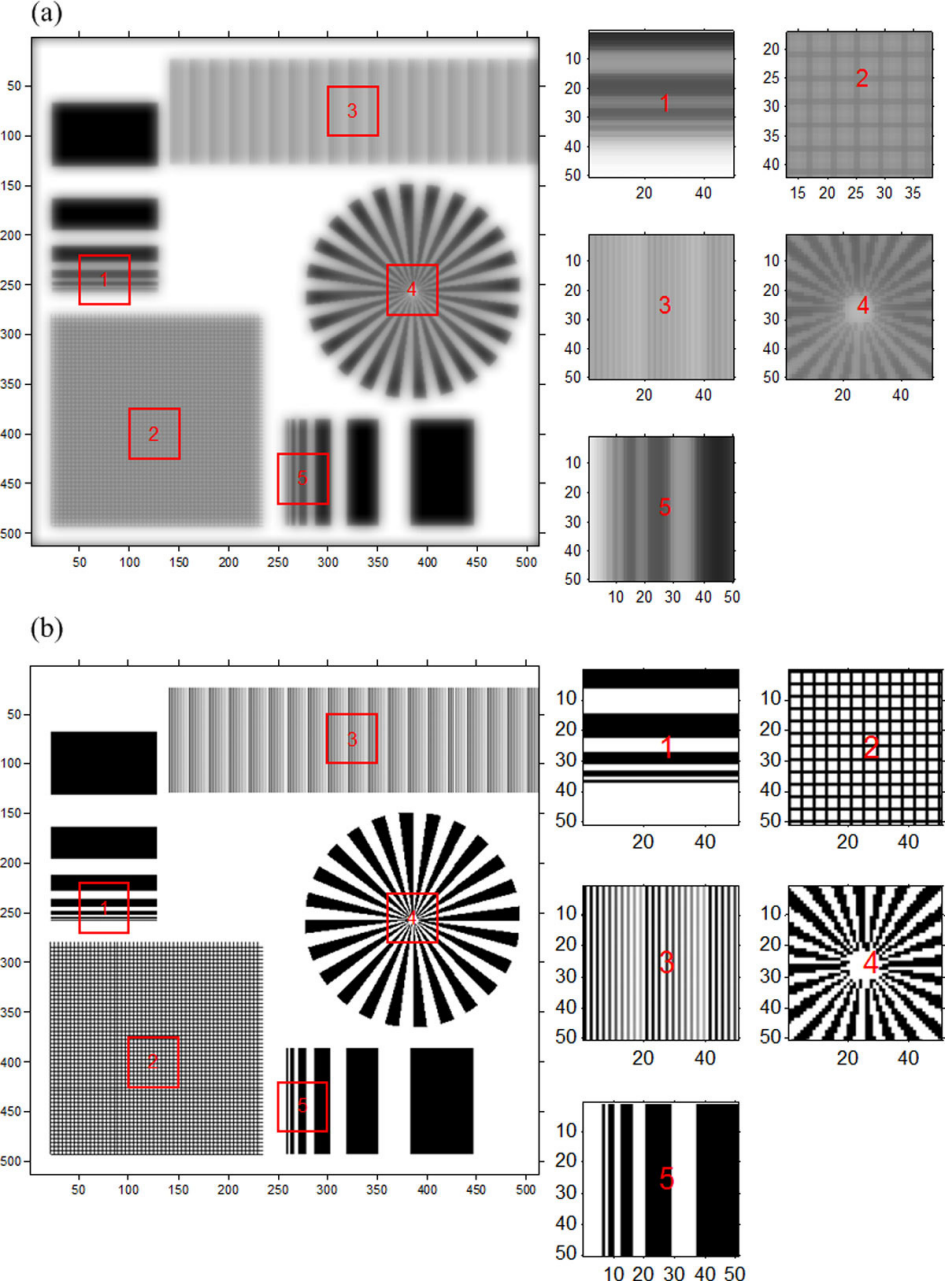
**Comparison between the blur image and the result images from the proposed method: (a) simulated obscure image and its some parts with magnification, (b) recovery image and some parts of it with magnification**.

In order to testify the effectiveness of the recovery algorithm, the complicated pattern shown in Figure 3 was used as the wanted high-resolution image to simulate the degraded blur image (Figure 4). After the blur image preparation, we first demonstrated the performance of the proposed recovery algorithm on the recovery of the noise-free degraded blur image. Further, Poisson noise was added to the degraded blur image to study the robustness of this algorithm. To evaluate the resolution of the restored image, modulation transfer function (MTF) [14] was brought in the experiment. And besides, quantifications based on signal to noise ratio (NSR) and peak signal to noise ratio (PNSR) were used to evaluate the quality of the restored image.

### Ideal projection data without noise

**Figure 5:**
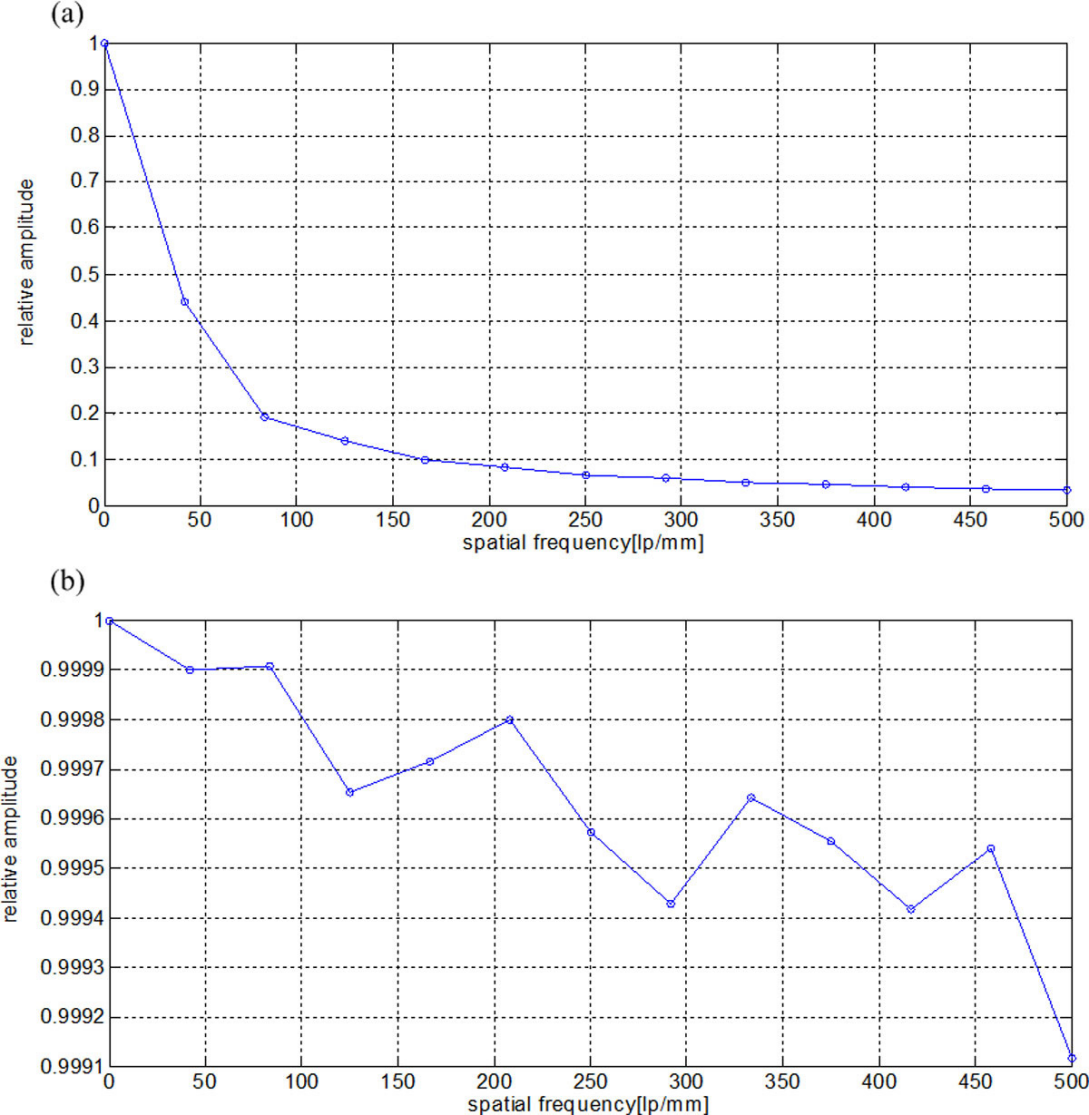
**Resolution comparison between the blur image and the result images from the proposed method: (a) MTF's for the optical system with 20 *μm *thick scintillator, (b) MTF's for the recovery image**.

We first tested the performance of the proposed algorithm on an ideal model, that is, a noise-free degraded projection image. The blur image together with some magnified parts of it is shown in Figure 4(a). From the blur image, we obtained a clear image (Figure 4(b)) by using the recovery algorithm proposed above. The MTF curve in Figure 5(a) shows that the spatial resolution of the blur image is only about 160 lp/mm according to 10% MTF. And the MTF in Figure 5(b) indicates that the recovery algorithm has perfect performance on the noise-free model, and it can make the optical system achieve a somewhat limited resolution.

### Projection data with noise

**Figure 6:**
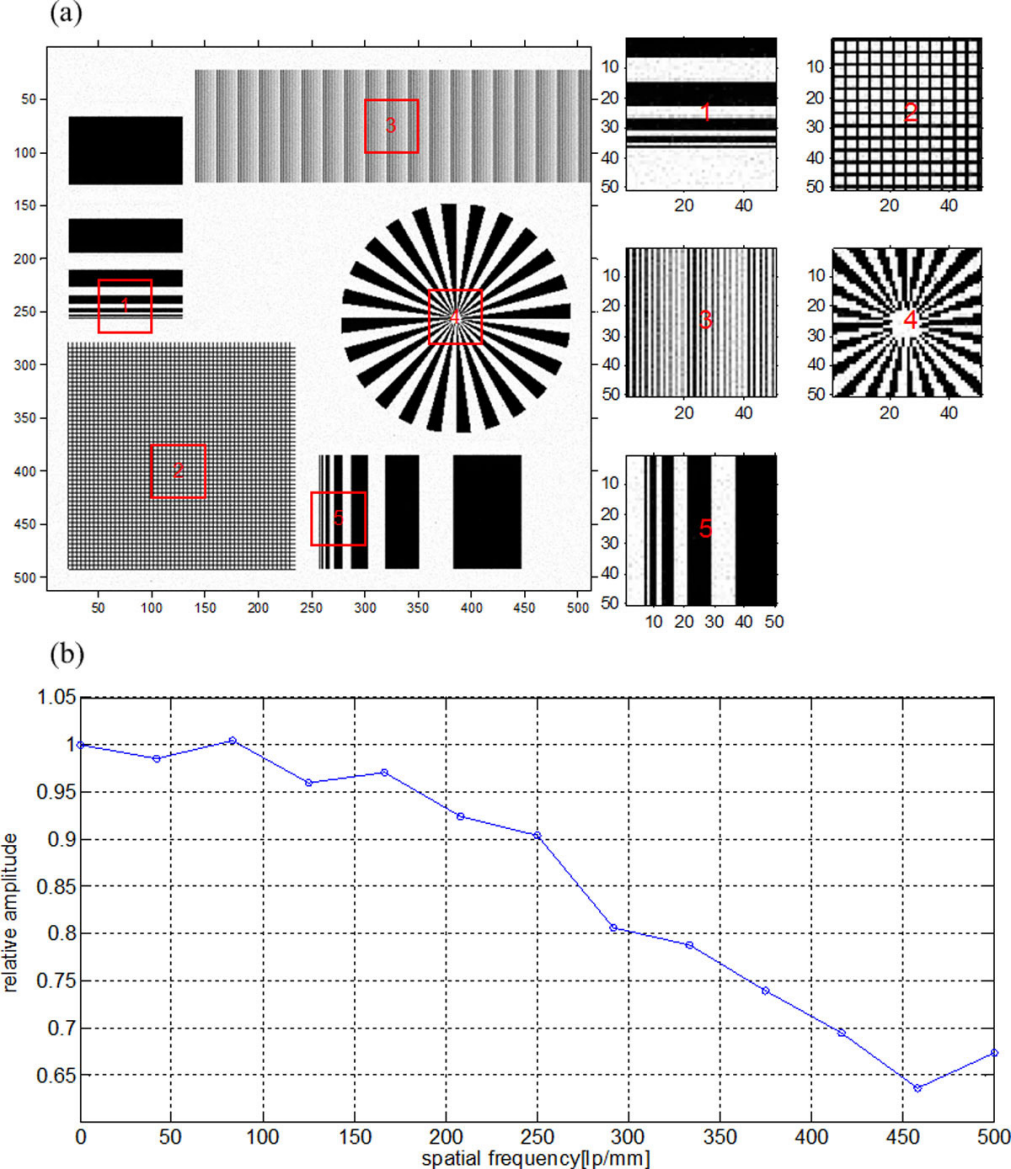
**The recovery image and its MTF curve when Poisson noise was added to the projection blur image: (a) recovery image from noisy projection data and some parts of it with magnification, (b) MTF's for the recovery image in (a)**.

From the experiment and analysis above, we can conclude that the proposed algorithm can perfectly recover the blur noise-free projection data. In the following experiment, we want to test whether this method is available under noisy condition. The noise having impact against recovery quality results from CCD camera. And the three primary sources of noise in a CCD imaging system are photon noise, dark noise, and read noise. Usually the involved CCD in the high-resolution X-ray imaging system is usually equipped with refrigeration unit to greatly reduce dark noise and utilizes design enhancement to reduce read noise, and besides, in practice the camera integration time is often increased to collect more photons and make photon noise exceed both dark noise and read noise, so the photon noise can be assumed the primary noise. The photon noise results from the inherent statistical variation in the arrival rate of photons incident on the CCD and the magnitude of signal containing it follows the Poisson statistical distribution of photons incident on the CCD at a given location. So in the experiment, we added Poisson noise to the noise-free blur projection image and we assumed the maximum transmitting photon number was 10^6^. From the recovery image in Figure 6(a) and the MTF curve in Figure 6(b), we can conclude that this algorithm can also recover the noisy blur image and achieve a somewhat limited resolution. But the only shortage is that this algorithm can not perfectly recover the low contrast parts (shown in part 3 of Figure 6(a)), and the quality of restored image depends on the signal to noise ratio of the degraded blur image.

### Quantification based evaluation

In addition to the visualization based evaluation above, we performed measurements of local SNR and PNSR to quantitatively evaluate the quality of the recovery image. The local SNR is defined as

$$\text{SNR = 20*log(}\frac{u}{\sigma }).$$

And, PNSR is computed as

$$\text{PSNR = 10*log}10\text{(}\frac{255^{2}}{\text{MSE}}).$$

**Figure 7:**
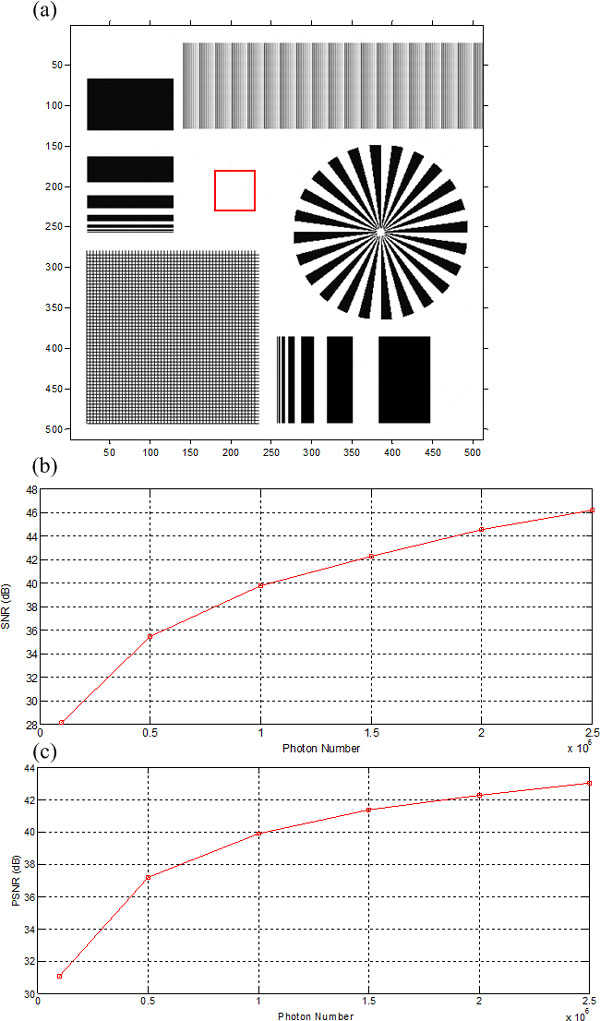
Quantitative quality evaluation of the recovery image for different levels of Poisson noise: (a) recovery image when incident photons number is 2.5 × 10^6^, (b) local SNR in the region encompassed by the square of (a) for different levels of noise, (c) PSNR for different levels of noise.

Here, MSE represents the Mean Square Error between the true image and the recovery image. In the experiment, several different levels of Poisson noise were added to the noise-free degraded blur image. From the recovery image in Figure 7(a) we can see that when the incident photon number reaches to 2.5 × 10^6^, the corresponding Poisson noise has nearly no impact against the quality of the recovery image. And from the local SNR and global PSNR curves for different noise level shown in Figure 7(b) (c), we can quantitatively determine how the noise level impacts the recovery image quality, so the curves can help us in practice to determine which noise level is suitable according to the quality requirement.

## Discussion

In this paper we simulated a luminous model of the scintillator which is thicker than the matching thickness. And based on this model, we developed a debluring algorithm to recover the blur image caused by defect of focus. From the analysis of MTF curves in the experiment, we demonstrated that the recovery algorithm can recover the blur image almost by a hundred percent and achieve a somewhat limited resolution. When the noise in real application is taken into consideration, this algorithm can also recover the blur image quite well, but the quality of the recovery image depends on the SNR level of the projection blur image acquired from CCD camera. That is to say, if we want to get a more accurate recovery image, we need higher SNR level of projection blur image. The stimulation in 3.3 can help us to choose which noise level is appropriate in a specific system. Of course, some techniques such as ML-EM algorithm, penalized weighted least-squares algorithm [15] which are respectively developed in PET and CT reconstruction may be modified here to suppress the noise and to improve the quality of the recovery image. And this modification is worthy for further study and discussion.

## Conclusion

The results in the experiment indicated that the proposed method could effectively extend the depth of high-resolution X-ray imaging system. And if we want to get a recovery image of higher quality, we should control the noise level of the degraded blur image. In order to put this algorithm in practice, we should also know the system constant c and calibrate the scintillator to make it to be perpendicular to the primary optical axis, and these are our future work.

## List of abbreviations used

CoC: Circle of confusion; DOF: Depth of focus; MTF: Modulation transfer function; NA: Numeric aperture; POCS: Projection onto convex sets; PSNR: Peak signal noise ratio; SNR: Signal noise ratio; TV: Total variation;

## Competing interests

The authors declare that they have no competing interests.

## Authors' contributions

GL and SL were responsible for the computational modeling and algorithm design. NG was responsible for the overall investigation and brought up a lot of valuable suggestions in the experiment. All authors have been involved in drafting the manuscript or revising it critically for important intellectual content and some details; and they have approved the final version for publication. Each author has participated sufficiently in the work to take responsibility for appropriate portion s of the content.

## Authors' information

Gu's group has been focusing on the research and application of all kinds of Nano-medical technologies including Micro or Nano CT imaging, nano-material, new nano-magnetic contrast agent and so on. For more information, please visit the website: http://lbmd.seu.edu.cn/nano/

Yan has been doing research in medical imaging and diagnosis for laryngeal disease and also doing research in single molecule imaging in cells and tissues. For more details, please see the website: http://www.scu.edu/engineering/bioengineering/yan.cfm

## Declarations

Publication of this article was paid with funding from Special Funds of the National Natural Science Foundation of China for Basic Research Projects of Scientific Instruments (Grant No.61127002)

This article has been published as part of *BioMedical Engineering OnLine *Volume 14 Supplement 1, 2015: Cardiovascular Disease and Vulnerable Plaque Biomechanics. The full contents of the supplement are available online at http://www.biomedical-engineering-online.com/supplements/14/S1

## References

[B1] Koch A Raven C Spanne P Snigirev A X-ray imaging with sub-micrometer resolution employing transparent luminescent screens J Opt Soc Am A 1998 15 1940 1950

[B2] Moosmann J Ershov A Altapova V X-ray phase-contrast in vivo microtomography probes new aspects of Xenopus gastrulation Nature 2013 497 374 377 23676755 10.1038/nature12116 PMC4220246

[B3] Ng R Levoy M Brédif M Duval G Horowitz M Hanrahan P Light Field Photography with a Hand-Held Plenopic Camera Technical Report CTSR 2005-02 2005 Dept. of Computer Science, Stanford Univ

[B4] Jang JY Shin D Kim ES Optical three-dimensional refocusing from elemental images based on a sifting property of the periodic δ-function array in integral-imaging Opt Express 2014 22 2 1533 1550 24515160 10.1364/OE.22.001533

[B5] Sezan MI PavloviC G Tekalp AM Erden AT On modeling the focus blur in image restoration IEEE International Conference on Acoustics, Speech, and Signal Processing 1991 2485 2487

[B6] Pentland AP A new sense for depth of field IEEE T Pattern Anal 1987 9 4 523 531 10.1109/tpami.1987.4767940 21869410

[B7] Subbarao M Surya G Depth from defocus: a spatial domain approach Int J Comput Vision 1994 13 3 271 294

[B8] Subbarao M Direct recovery of depth map I: differential methods IEEE Computer Society Workshop on Computer Vision, Miami Beach 1987

[B9] Kaczmarz S Angenaherte auflosung von systemem linearer gleichungen Bull Acad Pol Sci Lett A 1937 355 357

[B10] Candes E Romberg J Tao T Stable signal recovery from incomplete and inaccurate measurements Commun Pur Appl Math 2006 59 1207 1223

[B11] Sidky EY Pan XC Image reconstruction in circular cone-beam computed tomography by constrained, total-variation minimization Phys Med Biol 2008 53 4777 4807 18701771 10.1088/0031-9155/53/17/021 PMC2630711

[B12] Ritschl L Bergner F Fleischmann C Kachelrieß M Improved total variation-based CT image reconstruction applied to clinical data Phys Med Biol 2011 56 1545 1561 21325707 10.1088/0031-9155/56/6/003

[B13] Yu HY Wang G Compressed sensing based interior tomography Phys Med Biol 2009 54 2791 2805 19369711 10.1088/0031-9155/54/9/014 PMC2863332

[B14] Samei E Flynn MJ Reimann DA A method for measuring the presampled MTF of digital radiographic systems using an edge test device Med Phys 1998 25 1 102 113 9472832 10.1118/1.598165

[B15] Wang J Li TF Lu HB Penalized Weighted Least-Squares approach to sinogram noise reduction and image reconstruction for low-dose X-ray computed tomography IEEE T Med Imaging 2006 5 10 1272 1283 10.1109/42.896783 PMC1619874 17024831

