# Erratum to: A method of extending the depth of focus of the high-resolution X-ray imaging system employing optical lens and scintillator: a phantom study

**DOI:** 10.1186/s12938-016-0199-5

**Published:** 2016-07-04

**Authors:** Guang Li, Shouhua Luo, Yuling Yan, Ning Gu

**Affiliations:** School of Biological Science and Medical Engineering, Southeast University, Nanjing, 210009 China; Department of Bioengineering, School of Engineering, Santa Clara University, Santa Clara, CA 95053 USA

## Erratum to: BioMedical Engineering OnLine 2015, 14 (Suppl 1):S15 DOI 10.1186/1475-925X-14-S1-S15

After publication of this article [1], we became aware that the article type was erroneously given as ‘Review’, rather than ‘Research’. We apologise for this mistake.
